# EB virus infection-associated hemophagocytic lymphohistiocytosis complicated with giant axillary lymph nodes: A case report

**DOI:** 10.1097/MD.0000000000042608

**Published:** 2025-08-08

**Authors:** Chunyan Zhang, Chenda Feng, Wenjing Tang, Lichao Sun, Yuhang Mu

**Affiliations:** a Department of Rehabilitation Medicine, The First Hospital of Jilin University, Changchun, Jilin Province, People’s Republic of China; b Department of Emergency Medicine, The First Hospital of Jilin University, Changchun, Jilin Province, People’s Republic of China.

**Keywords:** Epstein–, Barr virus, giant axillary lymph nodes, hemophagocytic lymphohistiocytosis, shock

## Abstract

**Rationale:**

Hemophagocytic lymphohistiocytosis (HLH) is a rare, potentially fatal multisystem inflammatory disorder with nonspecific clinical manifestations. Early diagnosis of this condition is inherently challenging. Here, this study reports a case of hemophagocytic lymphohistiocytosis associated with Epstein–Barr virus (EBV) infection with giant axillary lymph nodes.

**Patient concerns:**

A 50-year-old woman presented with HLH who presented with fever and shock, followed by rapid impairment of liver and coagulation function, accompanied by giant lymph nodes in the right axilla.

**Diagnoses:**

Bone marrow examination revealed no abnormality. Epstein–Barr virus load of nucleic acid in peripheral blood was 1.65 × 10^6^ copies/mL. The number of EBV sequences detected by high-throughput sequencing was 543, which confirmed EBV infection.

**Interventions:**

We treat patients with symptomatic and supportive care.

**Outcomes:**

The patient responded to symptomatic and supportive treatment and was discharged. At 2-month follow-up, the patient had no complaints, and the EBV load of nucleic acid was <500 copies/mL.

**Lessons:**

In patients presenting with fever and shock followed by rapid impairment of liver function and coagulation function, and enlarged axillary lymph nodes, the possibility of EBV-HLH should be considered. Early identification and initiation of etiologic treatments for EBV-HLH are pivotal for good prognosis of patients.

## 1. Introduction

Hemophagocytic lymphohistiocytosis (HLH) is a rare, potentially fatal multisystem inflammatory condition that is often triggered by an underlying medical condition. HLH is characterized by excessive proliferation of activated lymphocytes and tissue cells, but ineffective immune response. The main symptoms are fever, hepatosplenomegaly, cytopenia, and elevated biomarkers of typical HLH, including ferritin and soluble interleukin-2 receptor.^[1,2]^ The condition has an acute onset and typically shows a rapidly progressive disease course. It is divided into familial HLH and secondary HLH. The most common form of secondary HLH is Epstein–Barr virus (EBV) infection-associated HLH (EBV-HLH).^[3]^ Both simple EBV infection and EBV-HLH can lead to multi-organ dysfunction. Due to the diverse and atypical symptoms of simple EBV infection, early diagnosis of EBV-HLH is typically challenging, and the condition is liable to be misdiagnosed as severe bacterial infection. We present a case of EBV-HLH who presented with fever and shock, followed by rapid impairment of liver function and coagulation function, accompanied by enlarged lymph nodes in the right axilla. Our experience with this case may improve the understanding of EBV-HLH and facilitate early diagnosis of this condition.

## 2. Case presentation

A 50-year-old woman was admitted to hospital on June 30, 2021 with chief complaints of intermittent fever for 4 days and decreased blood pressure for 1 day. The patient developed intermittent fever 4 days ago (peak temperature: 38.1°C) accompanied by chills. The fever responded to oral antipyretic and she received no further treatment. One day ago, she developed fatigue and subsequently became unconscious, and was brought to the emergency department of our hospital. Her past medical history was unremarkable. There was no history of smoking or alcohol use, or any exposure to COVID-19.

Her physical parameters at admission were: body temperature 36.7°C; pulse 110 beats/min; respiratory rate 20 per min; blood pressure 80/50 mm Hg. Her Glasgow Coma Scale score was 12 points (E3 V4 M5), accompanied by unconsciousness. Enlarged hard lymph nodes were palpable in the right axilla, with no tenderness or signs of adhesion to the surrounding tissues. No other abnormalities were detected on systemic examination.

**Figure 1.**
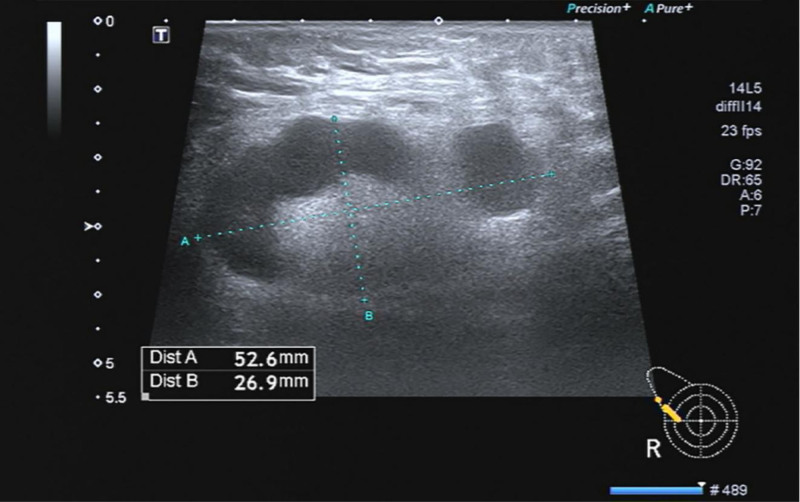
Ultrasound image showing giant lymph nodes. Superficial ultrasound revealed multiple enlarged axillary lymph nodes on both sides, and the largest lymph node was in the right axilla (size: 52.6 mm × 26.9 mm).

**Figure 2.**
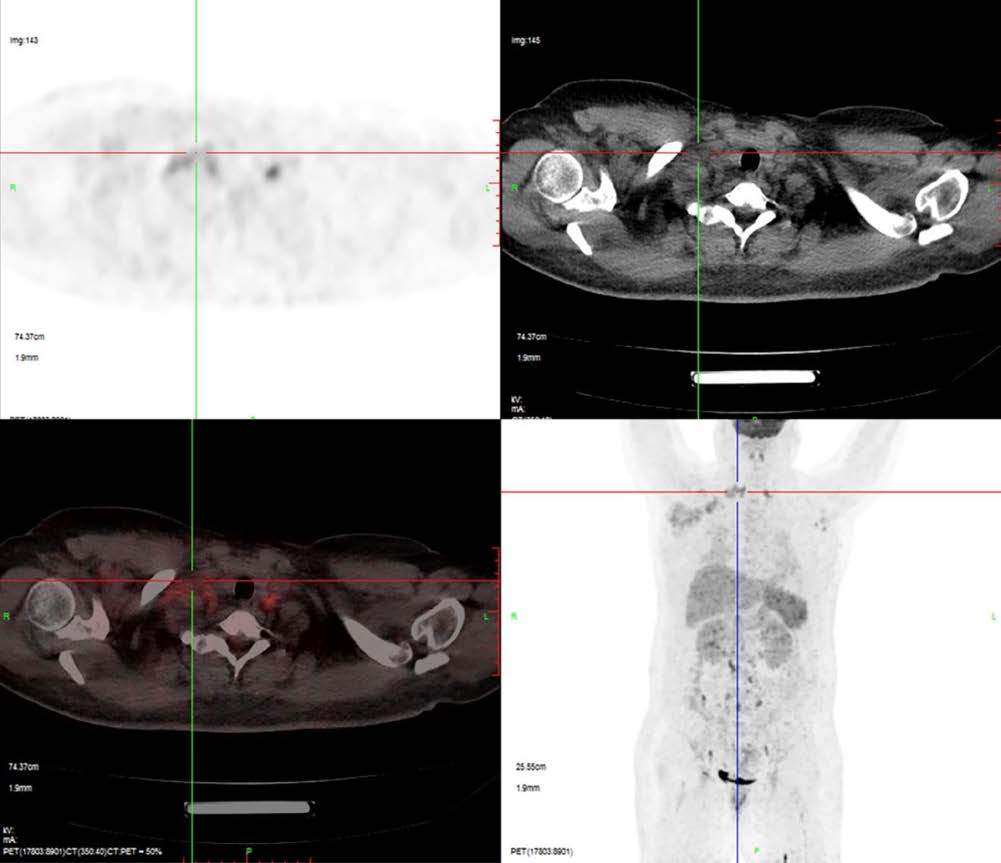
PET/CT images showing lymphomatous infiltration in liver and spleen. Whole-body positron emission tomography (PET)/CT showed that lymphoma could not be excluded, along with lymphomatous infiltration in liver and spleen. CT = computed tomography, PET = positron emission tomography.

About the auxiliary examinations, on admission (day 0), her laboratory parameters were as follows: white blood cell count 8.3 × 10^9^/L; platelet count 80 × 10^9^/L; serum aspartate transaminase 53.9 U/L; alanine aminotransferase 69.4 U/L; total bilirubin 7.7 μmol/L; direct bilirubin 2.5 μmol/L; activated partial thromboplastin time 32.8s; prothrombin time 12.2s; procalcitonin 0.27 ng/mL; and C-reactive protein 132.6 mg/L. Chest computed tomography (CT) revealed signs of mild inflammation in the right lung with right-sided pleural effusion, and lymphadenopathy in the right axilla. Four days after admission, EBV load was measured via real-time polymerase chain reaction (PCR) assay and the result was 1.65 × 10^6^ copies/mL. High-throughput sequencing revealed 543 sequences of EBV. Superficial ultrasound revealed multiple enlarged axillary lymph nodes on both sides, and the largest lymph node was in the right axilla (size: 52.6 mm × 26.9 mm; Fig. 1). Repeat chest CT showed bilateral pleural effusion and multiple enlarged lymph nodes in the right axilla and supraclavicular fossa. Abdominal CT scan showed multiple lymph nodes in the hilar hepatitis and slight enlargement of the mesentery. Other investigations, including sputum culture and fungal smear, serology for hemorrhagic fever, hepatitis A and E, quantification of hepatitis B virus, antinuclear antibody, anti-M2, acute flaccid paralysis, Immunoglobulin G of autoimmune hepatitis, protein electrophoresis, nucleic acid of cytomegalovirus, and anti-Brucella antibodies, showed no significant abnormality. Seven days after admission, her laboratory parameters were: significantly increased serum bilirubin level, deteriorated coagulation function (Table 1), serum triglyceride 1.51 mmol/L(−), ferritin 2913.3 μg/L(+), natural killer (NK) cell activity 21.69%(−), and soluble CD25 19,495 pg/mL(+). Histopathological examination of lymph node biopsy showed large areas of bleeding and necrosis, along with infiltration of acute and chronic inflammatory cells; lymphoid tissues were found in focal areas. Immunohistochemical results showed no signs of metastatic cancer, tuberculosis, or obvious invasive lymphoma in the examined tissues. Bone marrow puncture revealed hyperplastic anemia and poor megakaryocyte maturation. Whole-body positron emission tomography (PET)/CT showed that lymphoma could not be excluded, along with lymphomatous infiltration in liver and spleen (Fig. 2).

**Table 1:** Laboratory parameters after admission.

Days	WBC	HB	PLT	AST	ALT	TBIL	DBIL	APTT	PT	FBG	INR
	(10^9^/L)	(g/L)	(10^9^/L)	(U/L)	(U/L)	(μmol/L)	(μmol/L)	(s)	(s)	(g/L)	
Day 0	8.3	150.0	80.0	53.9	69.4	7.7	2.5	32.8	12.2	1.7	1.0
Day 1	22.7	158.0	71.0	387.0	294.2	24.0	12.1	–	–	–	–
Day 2	35.6	118.0	55.0	518.5	522.3	29.0	12.3	–	–	–	–
Day 3	39.8	117.0	46.0	496.0	561.9	43.7	12.8	–	–	–	–
Day 4	50.4	115.0	44.0	220.8	370.6	54.1	15.7	53.1	18.8	0.5	1.6
Day 5	42.7	95.0	32.0	105.1	186.8	65.4	22.1	50.2	15.2	0.9	1.3
Day 6	34.7	74.0	16.0	101.6	139.8	82.7	33.5	53.9	17.0	0.9	1.5
Day 7	16.4	66.0	9.0	83.8	79.7	85.7	35.2	61.4	16.2	0.6	1.4
Day 8	11.1	93.0	18.0	73.7	64.4	71.3	28.7	40.8	14.5	1.5	1.2
Day 9	7.2	62.0	22.0	48.6	37.7	58.7	21.3	46.6	13.6	1.4	1.3
Day 20	4.1	92.0	78.0	57.7	35.7	30.7	17.5	32.2	12.1	1.8	1.0

ALT = alanine aminotransferase, APTT = activated partial thromboplastin time, AST = aspartate transaminase, DBIL = direct bilirubin, FBG = fibrinogen, HB = hemoglobin, INR = international normalized ratio, PLT = platelet count, PT = prothrombin time, TBIL = total bilirubin, WBC = white blood cell.

About the diagnosis and treatment, based on the poor general condition of the patient at admission, medical history, clinical manifestations, and results of auxiliary examinations, a preliminary diagnosis of sepsis was made. The condition of the patient rapidly deteriorated despite prompt initiation of treatment including fluid resuscitation, antibiotics, and other supportive therapies, and she developed jaundice, edema, dyspnea, and abdominal distension, increased levels of infection markers, as well as acute impairment of liver function and coagulation function. Subsequently, EBV nucleic acid quantification, next generation sequencing (NGS) of peripheral blood, detection of typical HLH biomarkers, superficial ultrasound, PET/CT, bone marrow puncture, and other related investigations were performed. Symptomatic and supportive treatment included antiviral and hepatoprotective agents, blood transfusion, as well as thoracic puncture and drainage. Ten days after admission, all indicators showed a tendency for improvement. After consultation with a multidisciplinary team, the diagnosis of HLH was considered, and the treatment plan was continued. Twenty days after admission, all indicators had returned to normal and she was discharged. At 2-month follow-up, the patient had no complaints, and the EBV load of nucleic acid was <500 copies/mL. Immunoglobulin G of EBV Viral capsid antigens was positive (+) and IgM was negative (−). Ultrasound showed no change in the size of lymph nodes.

## 3. Discussion

EBV is classified as Herpes virus (type IV). It is one of the double-stranded deoxyribonucleic acid lymphotropic viruses, usually persisting in its human host as a latent asymptomatic infection. More than 95% of adults have positive serum EBV antibodies.^[4]^ EBV in childhood is usually asymptomatic or causes nonspecific symptoms; however, in adolescents and young adults, EBV infection is associated with HLH and infectious mononucleosis. Due to its acute onset, rapid progression along with diverse but nonspecific clinical manifestations, EBV-associated HLH is liable to be misdiagnosed as severe bacterial infection.

Our patient case was a middle-aged woman with intermittent fever and shock as the initial symptoms, and no obvious signs of pharyngeal inflammation at admission. Sequential organ failure assessment (SOFA) score has been proposed for the determination of sepsis.^[5]^ Apart from SOFA, some other new diagnostic markers are also useful in clinical practice.^[6]^ Our patient had a SOFA score of 3, which was indicative of high risk of sepsis. However, after active treatment for shock and antibiotics, the patient’s liver function and coagulation function continued to deteriorate. Finally, EBV infection was confirmed based on the results of PCR and NGS.

The diagnosis of HLH requires fulfillment of at least 5 of the following criteria: splenomegaly; fever; cytopenia in 2 or more cell lines in peripheral blood; hypertriglyceridemia and/or hypofibrinogenemia; identification of hemophagocytic cells in bone marrow, spleen, or lymph nodes; downregulation or complete absence of NK cell activity; hyperferritinemia; and increased level of soluble CD-25.^[1]^ Our patient qualified 5 diagnostic criteria (Nos. 2, 3, 4, 7, and 8), all of which supported the diagnosis of EBV-HLH. The initial treatment for EBV-HLH includes symptomatic and supportive therapy, as well as specific therapy. Specific therapy is aimed at cytotoxic control and immune regulation. According to a previous study,^[2]^ About >90% of patients can achieve remission after treatment with a 3-drug chemotherapy regimen including glucocorticoid, etoposide, and cyclosporine. In addition, intravenous immunoglobulin therapy, plasmapheresis, biotherapy, antitumor necrosis factor drugs, and even hematopoietic stem cell transplantation have shown to be effective for EBV-HLH.^[7]^ Based on this fact, we continued the treatment plan for this case, including antiviral, hepatoprotective, and supportive treatments, as advised by the hematologist.

More than 90% of patients with EBV infection show variable degrees of liver function impairment,^[8]^ mostly manifested by self-limiting elevation in the levels of alanine aminotransferase and aspartate transaminase, but obvious jaundice is rare.^[9]^ However, our patient showed severe liver function impairment. During the disease course, there was marked increase in bilirubin level, resulting in severe jaundice. The potential mechanism involves abnormal function of cytotoxic T cells and NK cells which either compromises the ability to clear infected cells, resulting in prolonged antigen presentation and ongoing stimulation of immune response, leading to hyperinflammatory response.^[3]^ Severe damage to liver function affects the synthesis of fibrinogen and coagulation factors. In our patient, the coagulation indices showed rapid deterioration before the treatment, resulting in severe coagulation impairment, mainly characterized by exogenous coagulation dysfunction. After treatment, the liver function and coagulation function were restored to normal and the patient was discharged.

EBV infection is usually accompanied by lymph node enlargement, and the morphological features of lymphatic tissue infected with EBV are similar to those of lymphoma.^[10]^ Tissue infiltration of activated T cells, antigen-presenting cells and macrophages, as well as excessive cytokine production, seems to contribute to the enlargement of lymph nodes.^[3]^ In this case, ultrasound showed multiple enlarged lymph nodes in her bilateral axilla, with the maximum size of 52.6 mm × 26.9 mm in the right axilla. Moreover, the PET/CT results did not exclude the possibility of lymphoma. In addition, lymphomatous infiltration of liver and spleen was detected. To identify the etiology, lymph node resection under general anesthesia was performed. Routine pathologic and immunohistochemical results showed extensive areas of hemorrhage and necrosis, accompanied by acute or chronic inflammatory cell infiltration; a few lymphatic tissues were found in focal areas; there were no signs of metastatic carcinoma, tuberculosis, or obvious aggressive lymphoma. These features were not in accordance with the diagnostic criteria of lymphoma.^[11]^ Ultrasonography of superficial lymph nodes 2 months later showed no change in the size of lymph nodes.

In this patient, acute EBV infection was confirmed by fluorescence quantitative PCR and NGS. NGS technology enables the indirect detection of a broad range of microorganisms, including viruses, bacteria, fungi and/or parasites, in clinical samples on the basis of uniquely identifiable deoxyribonucleic acid and/or ribonucleic acid sequences without requiring culture; their genetic composition and community function can be further studied based on genomics.^[12]^ NGS has been extensively applied for detection of the microbiome (bacteria, fungi, and viruses) in clinical samples owing to its distinct advantages in screening for underlying pathogens. Combined with clinical symptoms, it is a powerful diagnostic aid in clinical settings.

## 4. Conclusion

Above all, in patients presenting with fever and shock followed by rapid impairment of liver function and coagulation function, and enlarged axillary lymph nodes, the possibility of EBV-HLH should be considered. Multiple technologies should be adopted for etiological diagnosis as soon as possible in combination with the clinical manifestations. Early identification and initiation of etiologic treatments for EBV-HLH are pivotal for good prognosis of patients.

## Author contributions

**Data curation:** Wenjing Tang.

**Formal analysis:** Lichao Sun.

**Methodology:** Chenda Feng.

**Supervision:** Yuhang Mu.

**Writing – original draft:** Chunyan Zhang.
